# Efficacy of Telmisartan in MASLD/NAFLD: A Systematic Review and Meta‐Analysis of Randomized Controlled Trials

**DOI:** 10.1002/edm2.70319

**Published:** 2026-08-19

**Authors:** Shayan Shojaei, Dorsa Shekouh, Asma Mousavi, Salma Rostamipourfard, Saeed Karami, Salar Azadnik, Salma Nozhat, Seyed Alireza Mirhosseini, Erfan Hashemi, Noureen Fatima, Ali Fatehi Hassanabad, Mahsa Borjzadehgashtaseb, Alireza Arzhangzadeh

**Affiliations:** ^1^ Cardiovascular Diseases Research Institute Tehran University of Medical Sciences Tehran Iran; ^2^ Tehran Heart Center, Cardiovascular Diseases Research Institute Tehran University of Medical Sciences Tehran Iran; ^3^ Cardiovascular Research Center, School of Medicine Shiraz University of Medical Sciences Shiraz Iran; ^4^ Department of Internal Medicine Shiraz University of Medical Sciences Shiraz Iran; ^5^ Department of Cardiology, School of Medicine Shiraz University of Medical Sciences Shiraz Iran; ^6^ Department of Surgery University of Calgary Alberta Canada; ^7^ Department of Medicine Gujranwala Teaching Hospital Gujranwala Pakistan; ^8^ Department of Cardiac Sciences, Section of Cardiac Surgery, Libin Cardiovascular Institute University of Calgary, Cumming School of Medicine Calgary Alberta Canada; ^9^ Libin Cardiovascular Institute University of Calgary Calgary Alberta Canada; ^10^ Department of Surgery, Section of Cardiac Surgery, New York Presbyterian Hospital Columbia University Irving Medical Center New York City New York USA

**Keywords:** MASLD, meta‐analysis, telmisartan

## Abstract

**Background:**

Activation of the renin‐angiotensin‐aldosterone system contributes to hepatic inflammation in metabolic dysfunction‐associated steatotic liver disease (MASLD). Telmisartan, an angiotensin II receptor blocker with partial PPAR‐γ agonistic activity, has been hypothesized to confer metabolic benefits; however, current clinical evidence has not consistently demonstrated such effects.

**Aim:**

This systematic review and meta‐analysis evaluated the efficacy of telmisartan on metabolic, biochemical, and histological outcomes in MASLD.

**Methods:**

Electronic databases (PubMed, Embase, Web of Science, Scopus) were searched through 2026 for randomized controlled trials (RCTs) investigating telmisartan in adults with MASLD. Risk of bias was assessed using the RoB2 tool. Random‐effects meta‐analyses calculated pooled mean differences (MDs) with 95% confidence intervals (CIs).

**Results:**

Six RCTs comprising 258 participants (126 telmisartan, 132 controls) met the inclusion criteria. Telmisartan treatment was associated with a modest reduction in fasting blood sugar compared with controls (MD = −2.78 mg/dL; 95% CI −5.17 to −0.39; *p* = 0.023); however, this finding was accompanied by substantial heterogeneity (I^2^ = 91.8%) and should therefore be interpreted cautiously. No significant changes were observed for body mass index, waist circumference, HOMA‐IR, lipid profiles, ALT, or AST. Conversely, gamma‐glutamyl transferase (GGT) levels showed a modest but significant increase with telmisartan (MD = 5.40 U/L; 95% CI 0.51 to 10.29; *p* = 0.031). Histological analyses revealed a non‐significant trend toward fibrosis stage improvement (MD = −0.28; 95% CI −0.57 to 0.01; *p* = 0.060), with no significant improvements seen in the overall NAFLD activity score, steatosis, ballooning, or lobular inflammation. Most included trials carried a low overall risk of bias.

**Conclusion:**

Current evidence does not demonstrate consistent metabolic, biochemical, or histological benefits of telmisartan in MASLD. Although a modest reduction in fasting blood glucose was observed, substantial between‐study heterogeneity and the modest effect size limit confidence in this finding.

## Graphical abstract

Evidence from six randomized controlled trials indicates that telmisartan offers modest glycemic benefit but does not consistently improve metabolic, biochemical, or histological outcomes in MASLD/NAFLD, highlighting the need for larger, well‐designed clinical trials.

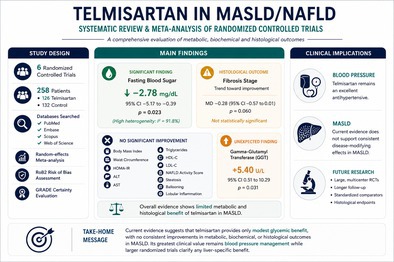

## 1 Introduction

Metabolic dysfunction‐associated steatotic liver disease (MASLD), formerly known as non‐alcoholic fatty liver disease (NAFLD), is one of the most common liver diseases worldwide, affecting roughly one in four adults [1, 2]. It ranges from simple fat accumulation in the liver to a more severe form called non‐alcoholic steatohepatitis (NASH), which can progress to liver scarring, cirrhosis, and hepatocellular carcinoma [3]. MASLD is closely linked to obesity, Type 2 diabetes, high blood pressure and high cholesterol. In fact, the leading cause of death in MASLD patients is not liver failure but cardiovascular disease, which highlights the need for treatments that address both the liver and the broader metabolic picture [4, 5, 6].

Insulin resistance lies at the core of MASLD, increasing hepatic fat production and triggering inflammation, oxidative stress, and hepatocyte injury [7]. Concurrent activation of the renin‐angiotensin system (RAAS) also plays an important role in worsening liver damage. A key hormone in this system, angiotensin II, promotes hepatic stellate cell activation, release of pro‐inflammatory cytokines and reactive oxygen species, and ultimately fibrosis through its Type 1 receptor (AT1R) [8, 9]. Blocking this receptor has therefore been proposed as a useful strategy to slow MASLD progression [10].

Telmisartan is a long‐acting angiotensin II receptor blocker (ARB) that differs from other drugs in its class because it partially activates peroxisome proliferator‐activated receptors γ (PPAR‐γ) and α (PPAR‐α), which regulate insulin sensitivity and lipid metabolism [11, 12]. This dual pharmacological profile has led to the hypothesis that telmisartan may improve both hepatic and metabolic dysfunction in MASLD. However, whether these theoretical advantages translate into clinically meaningful benefits remains uncertain [13].

Several small clinical trials have tested telmisartan in MASLD (historically classified as NAFLD) patients, measuring its effects on liver enzymes, blood sugar, cholesterol, body weight and liver histology. However, these trials included small numbers of patients, used different comparators, and reported mixed results [14, 15, 16]. Consequently, it remains uncertain whether telmisartan provides clinically meaningful benefits in patients with MASLD [10, 17]. Although a prior meta‐analysis examined angiotensin receptor blockers broadly in NAFLD, it did not focus exclusively on telmisartan, did not incorporate histological outcomes such as liver fat quantification, inflammation grading, and fibrosis staging, and was conducted before the adoption of the current MASLD diagnostic nomenclature and criteria [17]. To our knowledge, this is the first meta‐analysis to focus exclusively on telmisartan in MASLD, covering both metabolic and biopsy‐confirmed histological outcomes within the framework of current diagnostic criteria.

We conducted a pre‐registered systematic review and meta‐analysis of randomized controlled trials evaluating telmisartan in adults with MASLD, following the PRISMA 2020 reporting guidelines [18]. By pooling data from these trials, our primary objective was to determine whether telmisartan improves histological liver outcomes, including resolution of NASH, reduction in the NAFLD activity score and improvement in fibrosis stage. Secondary objectives were to assess effects on imaging markers of steatosis (MRI‐PDFF, CT liver‐to‐spleen ratio, CAP), non‐invasive fibrosis measures (transient elastography, serum fibrosis scores), liver biochemistry (ALT, AST, GGT), metabolic outcomes (insulin resistance, weight, blood pressure), and adverse events. These findings are intended to inform clinical decision‐making for patients with MASLD requiring antihypertensive therapy and to identify research priorities for future trials.

## 2 Methods

This study was conducted utilizing the Preferred Reporting Items for Systematic Reviews and Meta‐Analyses (PRISMA) guidelines [18]. The protocol of this meta‐analysis was registered on the Prospective Register of Systematic Reviews (PROSPERO). The full registration citation is: Efficacy of telmisartan in non‐alcoholic fatty liver disease: A systematic review and meta‐analysis of randomized trials. PROSPERO 2025 CRD420251132852.

### 2.1 Search Strategy

We conducted a systematic search through different databases, including PubMed, Embase, Cochrane, and Scopus, evaluating all available studies from inception to 2026. We designed our search strategy based on certain key terms, such as ‘Telmisartan’ OR ‘Angiotensin receptor blocker’ OR ‘ARBs’ AND ‘MASLD’ OR ‘NAFLD’ OR ‘Hepatic Steatosis’ OR ‘non‐alcoholic fatty liver disease’ OR ‘Fatty Liver’ AND ‘Metabolic syndrome’ OR ‘Insulin Resistance’ OR ‘Glucose metabolism’ OR ‘Dyslipidemia’ with their synonyms and equivalent terms, to be as broad and comprehensive as possible. No additional restrictions were applied. The detailed search strategy is demonstrated in Table S1. Subsequently, we conducted an additional manual search through similar studies and the reference list of included articles to cover any missing articles from our screening process. Grey literature sources, including ClinicalTrials.gov and WHO International Clinical Trials Registry Platform (ICTRP), were not searched, which represents a limitation acknowledged in the Limitations section.

### 2.2 Eligibility Criteria and Screening

This meta‐analysis included randomized controlled trials (RCTs) evaluating the efficacy of telmisartan therapy on metabolic, biochemical, and histological liver outcomes in patients with MASLD/NAFLD. We excluded reviews, conference abstracts, letters to the editor, editorials, case reports, and animal studies. Studies published in languages other than English were also excluded. After removing duplicates, two authors independently screened the titles and abstracts of the gathered articles according to the predefined eligibility criteria. Remaining studies were assessed based on full texts to select included studies by the aforementioned reviewers. Any discrepancies between the reviewers were resolved by the third expert reviewer. Throughout this review, MASLD is used as the preferred terminology when referring to the disease in general. However, the original terms NAFLD and NASH are retained when referring to the included studies, their eligibility criteria, and established histological scoring systems, as these trials predated the 2023 nomenclature change. The 2023 multi‐society Delphi consensus confirmed that both terms describe largely the same patient population, and studies enrolled under NAFLD/NASH criteria were therefore considered eligible as they broadly satisfy current MASLD diagnostic criteria.

### 2.3 Data Extraction

The data extraction process was conducted by two independent authors (DS and SR). The collected data included study characteristics, patient demographics, intervention details and outcomes. For each outcome of interest, data were collected for baseline and post‐intervention measurements, as well as the change between these time points when available. A third reviewer (AA) resolved any disagreements that occurred during the extraction.

### 2.4 Risk of Bias Assessment

The quality and risk of bias of the included studies were independently assessed using RoB2 instructions by two reviewers (SK And SA), with discrepancies resolved by consulting with a senior author (AA).

### 2.5 Certainty of Evidence Assessment

The certainty of evidence for critical outcomes was assessed using the grading of recommendations assessment, development and evaluation (GRADE) framework. Evidence derived from randomized controlled trials was initially rated as high certainty and downgraded according to risk of bias, inconsistency, indirectness, imprecision and publication bias.

### 2.6 Statistical Analysis

The primary objective of the current meta‐analysis was to assess the comparative changes of telmisartan versus other management approaches in MASLD. Due to the continuous nature of the measurement scales for comparison from the baseline to the endpoint of the studies, the mean differences (MDs) with their 95% confidence intervals (95% CIs) in the Telmisartan group compared to the comparator group obtained from each study were considered the main effect size for the pooled analyses. Where studies primarily provided the mean changes from baseline to endpoint, with their corresponding standard deviations (SDs) for each telmisartan and comparator group, the values were directly imported into the meta‐analysis. Where each mean difference or its corresponding SD for the telmisartan or comparator group was not reported, the values were manually calculated from the pre‐ and post‐data according to the Cochrane guidelines for systematic reviews and meta‐analyses [19]. We asked the corresponding author of the study for additional information in cases where manual calculation was impossible. Given the heterogeneous nature of the effect sizes, a random‐effects model was adopted as the appropriate analytical framework for the pooled analyses, reflecting anticipated clinical and methodological heterogeneity across the finally included studies. Clinical heterogeneity was anticipated because comparator interventions differed across studies and included placebo, lifestyle modification, vitamin E, losartan, valsartan, and orlistat‐containing regimens. Because only a small number of studies were available within each comparator category, comparator‐specific subgroup analyses and meta‐regression were not considered statistically appropriate.

For estimating the between‐study variance (τ^2^), the restricted maximum likelihood (REML) method was utilized. R software (R Foundation for Statistical Computing, Vienna, Austria), version 4.5.1, was used for all analyses, utilising the meta and metafor packages. Meta‐analyses were conducted individually for each outcome. Statistical heterogeneity was assessed using the I^2^ and Tau^2^ (τ^2^), with I^2^ values of 25%, 50%, and 75% indicating low, moderate, and substantial heterogeneity, respectively. For each outcome with high heterogeneity (I^2^ > 75%), leave‐one‐out sensitivity analyses were conducted and provided in the supporting information section. A *p*‐value of less than 0.05 was considered statistically significant.

Subgroup analyses were conducted to explore the effects of telmisartan by categorising outcome measures into three main groups: Body composition (BMI and WC); laboratory data (HOMA‐IR, FBS, LDL, TG, HDL, AST, ALT and GGT); and histological changes (fibrosis score, NAS score, and its components: ballooning, lobular inflammation and steatosis). Although meta‐regression was pre‐specified in the study protocol, it was ultimately not performed because only six randomized controlled trials were available, precluding a reliable regression‐based assessment of potential effect modifiers. Due to the potential for inflated Type 1 error in multiple comparative analyses, all subgroup analyses should be interpreted as exploratory.

Publication bias was assessed using funnel plots for outcomes; however, formal statistical tests for funnel plot asymmetry, including Egger's regression test and Begg's rank correlation test, were not performed, given the small number of included studies for each outcome. In instances of suspected visual asymmetry, the trim‐and‐fill approach was utilised as an approach to impute potentially missing studies and to provide bias‐adjusted pooled value estimates.

## 3 Results

**FIGURE 1:**
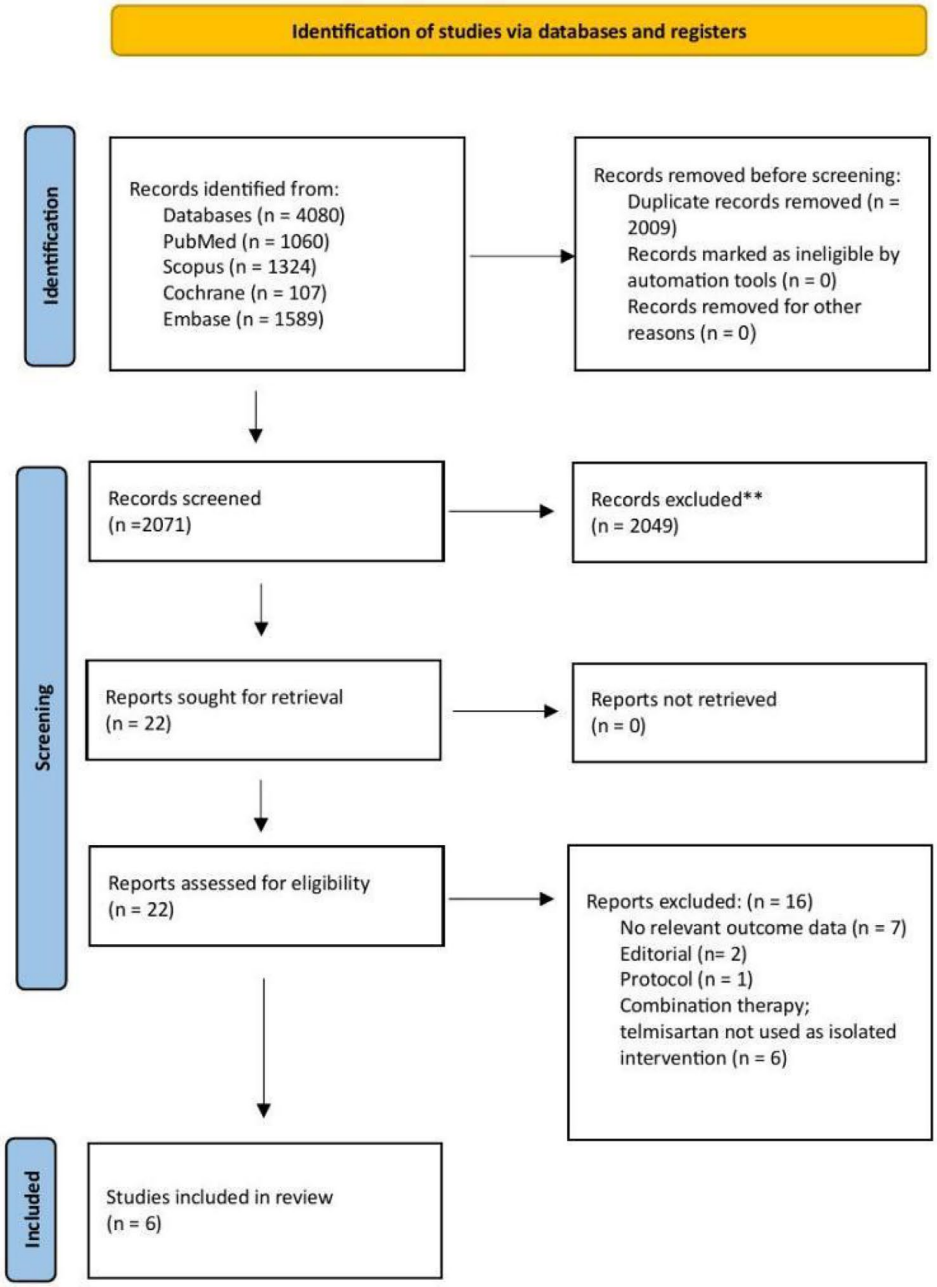
PRISMA flow chart depicting the study selection process.

We identified 4080 studies through various databases (PubMed, Embase, Scopus, chochrane). After removing duplicates and excluding studies based on title/abstract screening, 22 studies underwent full‐text evaluation. After the final exclusion, we included six studies [14, 15, 16, 20, 21]. The screening process is demonstrated in the PRISMA flow chart (Figure 1).

This meta‐analysis included six RCTs [14, 15, 16, 20, 21], comprising 258 participants: 126 (48.84%) in the telmisartan group and 132 (51.16%) in the control group. Follow‐up durations ranged from 2 weeks to 20 months. The overall mean (±SD) age was 44.84 (±9.91) years, with 40.70% male participants. Baseline characteristics of the included studies are summarized in Table 1.

**TABLE 1:** Baseline characteristics of included studies.

Study characteristics									Case (Telmisartan)			Control		
First author/Publication year	Country	Follow‐up duration	Age total (mean ± SD)	% male total	Case trait	Control trait	Sample size total	Adverse event	N	Age (mean ± SD)	% male	N	Age (mean ± SD)	% male
VAW Esmail2022 [19]	Iraq	8–12 weeks	43.3 ± 9.0	39.2	20 mg/day Telmisartan	120 mg/kg Orlistat/Carboxymethyl cellulose^b^	49	Not reported	24	NA	NA	25/25^b^	NA/NA^b^	NA/NA^b^
S Alam [15]	Bangladesh	12 months	37.9 ± 8.4	33.3	40 mg/day^a^ Telmisartan + life style modification	800 IU Vitamin E + life style modification	30	Not reported	16	36.6 ± 7.8	31.3	14	39.5 ± 9.0	35.7
MSM Al‐Nimer [20]	Iraq	8 weeks	43.6 ± 9.3	43.1	20 mg/day Telmisartan	Dummy tablet	51	No Significant adverse reactions were reported	26	43.3 ± 9.0	57.7	25	44.0 ± 9.7	28
S Alam [14]	Bangladesh	12 months	41.5 ± 10.5	25.8	40 mg/day Telmisartan + life style modification	Life style modification	30	Mild headache, dizziness, and abdominal pain were reported at similar frequencies in both groups. No cases of hypotension occurred in the telmisartan group, and no participants discontinued treatment because of adverse events	20	43.3 ± 11.0	20	10	37.9 ± 8.7	30
T Hirata [16]	Japan	12 months	58.6 ± 13.2	47.4	20 mg/day + dietary instructions	50 mg/day Losartan + dietary instructions	19	No participant discontinued the trial because of adverse events	12	57.7 ± 12.8	50	7	60.3 ± 14.3	42.9
EF Georgescu [21]	Romania	20 months	48.9 ± 1.4	51.8	20 mg/day Telmisartan	80 mg/day Valsartan	54	Not reported	28	48 ± 2.0	50	26	49.9 ± 2.1	46.2

Abbreviations: IU, international unit; kg, kilogram; mg, milligram; *N*, number; NA, not available; SD, standard deviation.

^a^ Doses were elevated to 80 mg/day if hypertension persisted after 40 mg of telmisartan.

^b^ The study included two control groups; data for each group are separated by ‘/’.

### 3.1 Quality Assessment

Risk of bias in the included studies was assessed using the RoB2 tool. Four of the six studies showed low risk of bias overall. The remaining two studies raised some concerns: S. Alam [15] in Domain 1 and T. Hirata [16] in Domain 2, as detailed in Table S2.

### 3.2 Outcomes

We evaluated differences in changes from baseline to after the treatment between case and control groups across all included studies. Detailed results for each outcome are presented in Table 2.

**TABLE 2:** Meta‐analysis of metabolic and histological outcomes.

Outcome variable type	Variable	N. study	Test of association		Test of heterogeneity		
			Mean difference (95% CI)	*p*	*p*	I^2^	Tau^2^
Body composition parameter	BMI	6	0.23 (−0.07 to 0.53)	0.138	0.201	29.7%	0.045
	WC	5	−1.07 (−3.68 to 1.54)	0.423	0.875	0%	0
Laboratory data	HOMA‐IR	4	−0.40 (−1.43 to 0.63)	0.450	< 0.001	86.1%	0.850
	FBS	5	−2.78 (−5.17 to −0.39)	0.023	< 0.001	91.8%	7.044
	LDL	2	−17.59 (−46.33 to 11.14)	0.230	0.247	25.5%	130.346
	TG	6	−2.46 (−16.86 to 11.94)	0.738	0.762	0%	0
	HDL	5	−1.33 (−3.31 to 0.65)	0.190	0.950	0%	0
	ALT	5	−1.37 (−6.72 to 3.98)	0.616	0.473	0%	4.250
	AST	4	−0.80 (−5.28 to 3.67)	0.726	0.537	0%	0
	GGT	4	5.40 (0.51 to 10.29)	0.031	0.849	0%	0
Histological outcomes	Fibrosis	3	−0.28 (−0.57 to 0.01)	0.060	0.466	0%	0
	NAS	3	−0.38 (−1.70 to 0.94)	0.571	< 0.001	88.8%	1.220
	NAS steatosis	2	0.21 (−0.54 to 0.95)	0.586	0.013	84.0%	0.243
	NAS ballooning	3	−0.21 (−0.46 to 0.05)	0.115	0.440	0%	0
	NAS lobular inflammation	2	−0.12 (−0.74 to 0.51)	0.715	0.003	88.5%	0.181

Abbreviations: ALT, alanine aminotransferase; AST, aspartate aminotransferase; BMI, body mass index; CI, confidence interval; FBS, fasting blood sugar; GGT, gamma‐glutamyl transferase; HDL, high‐density lipoprotein cholesterol; HOMA‐IR, homeostasis model assessment of insulin resistance; LDL, low‐density lipoprotein cholesterol; *N*, number of; NAS, non‐alcoholic fatty liver disease activity score; SD, standard deviation; TG, triglycerides; WC, waist circumference; τ^2^ (Tau^2^), between‐study variance in random‐effects meta‐analysis.

#### 3.2.1 Body Composition Parameters

##### 3.2.1.1 BMI

**FIGURE 2:**
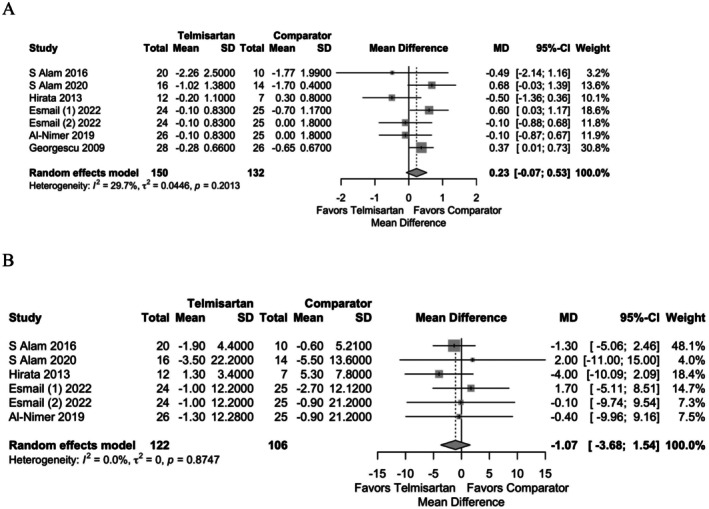
Forest plots for body composition outcomes: (A) BMI and (B) waist circumference.

A total of six studies were included in the analysis of BMI changes. The pooled analysis demonstrated no significant difference between case and control groups (MD = 0.23; 95% CI −0.07 to 0.53; *p* = 0.138). It also demonstrates that heterogeneity was low (I^2^ = 29.7%), and between‐study variance was minimal (Tau^2^ = 0.045) (Figure 2A).

##### 3.2.1.2 WC

Five studies evaluated WC, showing no significant reduction with telmisartan (MD = −1.07; 95% CI −3.68 to 1.54; *p* = 0.423). Heterogeneity and between‐study variance were negligible (I^2^ = 0% and Tau^2^ = 0) (Figure 2B).

#### 3.2.2 Laboratory Data

##### 3.2.2.1 HOMA‐IR

**FIGURE 3:**
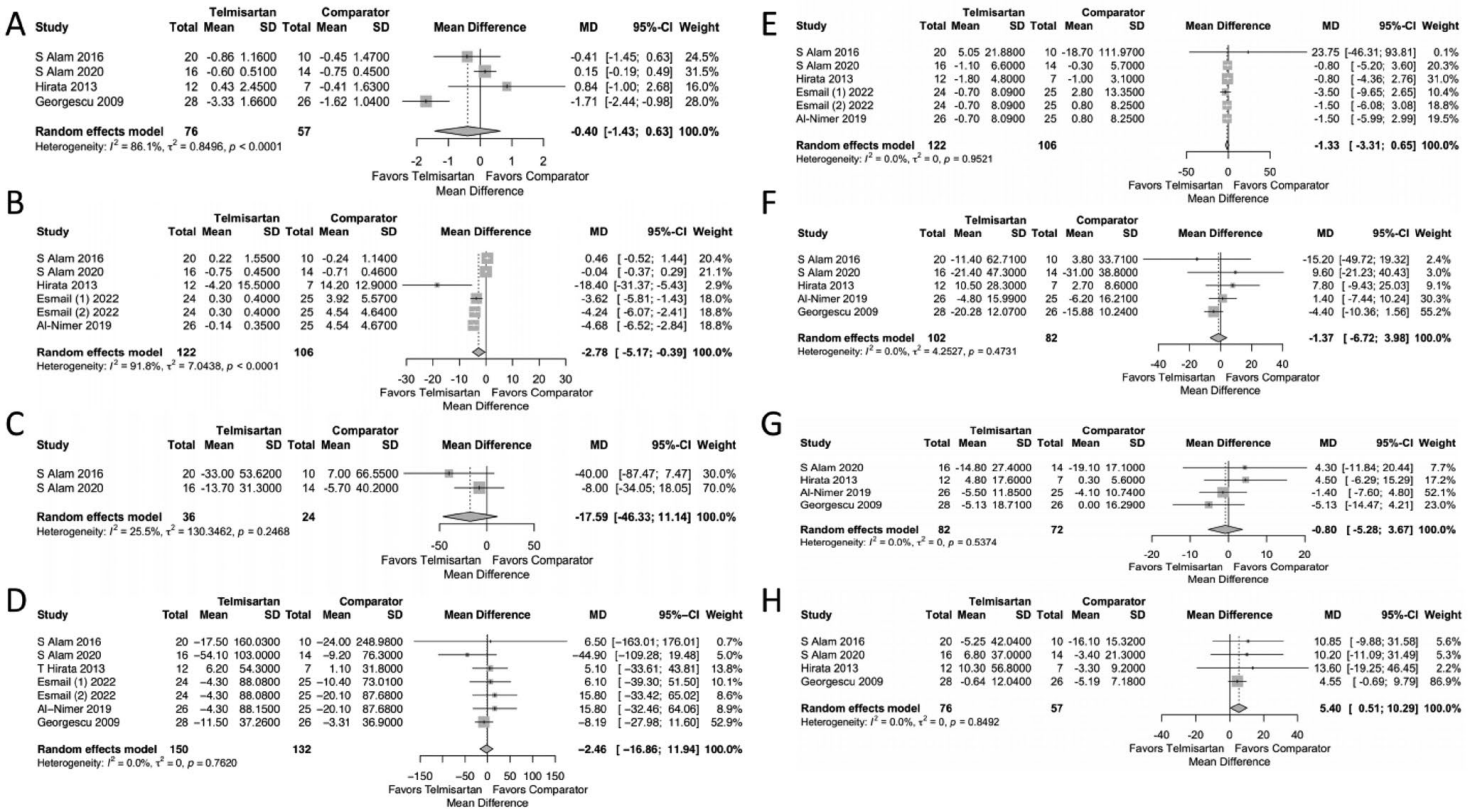
Forest plots for laboratory outcomes: (A) HOMA‐IR, (B) fasting blood sugar, (C) LDL, (D) triglycerides, (E) HDL, (F) ALT, (G) AST, (H) GGT.

HOMA‐IR was evaluated in four studies and showed no significant improvement in the telmisartan group compared to the control group (MD = −0.40; 95% CI −1.43 to 0.63; *p* = 0.450). Considerable heterogeneity and variance were present in this analysis (I^2^ = 86.1% and Tau^2^ = 0.850) (Figure 3A).

##### 3.2.2.2 FBS

Given the substantial heterogeneity (I^2^ = 91.8%), this finding should be interpreted cautiously despite achieving statistical significance (MD = −2.78; 95% CI −5.17 to −0.39; *p* = 0.023) (Figure 3B). The observed variability may reflect differences among the included studies in comparator interventions, follow‐up duration, and patient characteristics.

##### 3.2.2.3 Lipid Profile

Lipid parameters showed no significant changes across groups. Two studies on LDL showed a non‐significant decrease (MD = −17.59; 95% CI −46.33 to 11.14; *p* = 0.230) with low heterogeneity (I^2^ = 25.5%) (Figure 3C). Analyses regarding TG reported in six studies, and HDL reported in five studies, also demonstrated no significant differences (MD = −2.46, 95% CI −16.86 to 11.94; *p* = 0.738) and (MD = −1.33, 95% CI −3.31 to 0.65; *p* = 0.190). The heterogeneity for both outcomes was negligible (I^2^ = 0%, for both) (Figure 3D,E).

##### 3.2.2.4 Liver Enzymes

For liver enzymes, ALT (five studies) and AST (four studies) did not show significant improvements (MD = −1.37; 95% CI −6.72 to 3.98; *p* = 0.616) and (MD = −0.80; 95% CI −5.28 to 3.67; *p* = 0.726), both with narrow heterogeneity (I^2^ = 0%) (Figure 3F,G). GGT changes from four studies showed a significant increase with telmisartan (MD = 5.40; 95% CI 0.51 to 10.29; *p* = 0.031) (Figure 3H).

#### 3.2.3 Histological Outcomes

##### 3.2.3.1 Fibrosis

**FIGURE 4:**
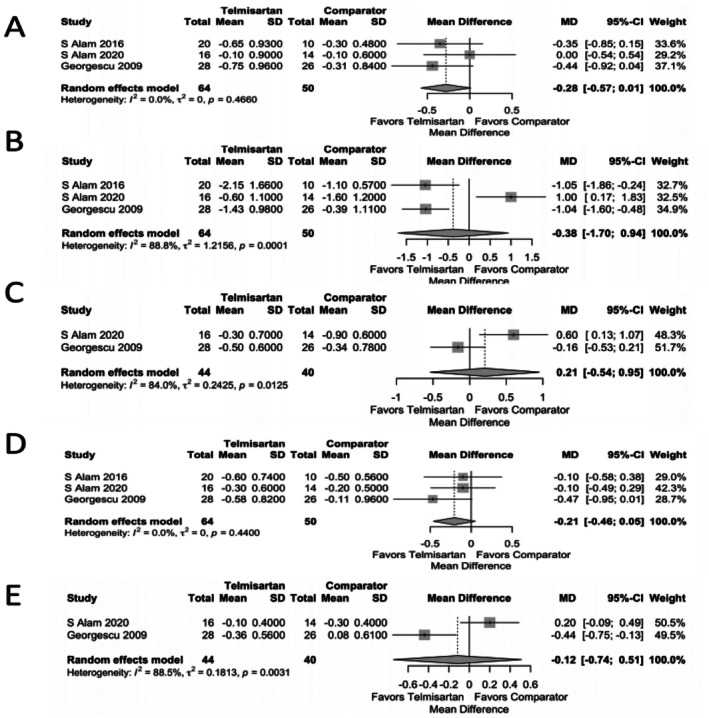
Forest plots for histological outcomes: (A) fibrosis stage, (B) NAFLD activity score (NAS), (C) NAS steatosis, (D) NAS ballooning, and (E) NAS lobular inflammation.

We included three studies reporting fibrosis stage changes. Three studies reported fibrosis stage changes. The pooled analysis showed no statistically significant difference between groups (MD = −0.28; 95% CI −0.57 to 0.01; *p* = 0.060), with no observed heterogeneity (I^2^ = 0%) (Figure 4A).

##### 3.2.3.2 NAFLD Activity Score (NAS)

Three studies on overall NAS showed no significant difference (MD = −0.38; 95% CI −1.70 to 0.94; *p* = 0.571). High heterogeneity was observed (I^2^ = 88.8%) in this analysis (Figure 4B). Two studies evaluated NAS‐steatosis. The pooled difference was not significant comparing case and control groups (MD = 0.21; 95% CI −0.54 to 0.95; *p* = 0.586), with substantial heterogeneity (I^2^ = 84.0%) (Figure 4C). NAS‐ballooning (three studies) also demonstrated a non‐significant improvement in the case group with no heterogeneity (MD = −0.21; 95% CI −0.46 to 0.05; *p* = 0.115; I^2^ = 0%) (Figure 4D). NAS‐lobular inflammation (two studies) yielded a similarly non‐significant difference (MD = −0.12; 95% CI −0.74 to 0.51; *p* = 0.715). However, the observed heterogeneity was high (I^2^ = 88.5%) (Figure 4E).

### 3.3 Certainty of Evidence

According to the GRADE framework, certainty of evidence ranged from low to moderate across evaluated outcomes. Evidence certainty was downgraded primarily because of substantial heterogeneity and imprecision related to the limited number of available studies. Detailed GRADE assessments are presented in Table S3.

### 3.4 Sensitivity Analysis

Due to high heterogeneity in the HOMA‐IR, FBS, and NAS analyses (I^2^ > 70%), as well as the unexpected statistically significant increase in GGT, leave‐one‐out sensitivity analyses were performed to evaluate the robustness of the pooled estimates. For HOMA‐IR, sequential exclusion of individual studies altered the magnitude of the pooled effect but not its direction, indicating that no single study explained the observed heterogeneity (Figure S1). For FBS, exclusion of the studies by S. Alam et al. [15], S. Alam et al. [14], or Hirata et al. [16] resulted in statistically significant between‐group differences (Figure S2). For NAS, exclusion of the S. Alam et al. [14] study eliminated heterogeneity (I^2^ = 0%) and yielded a statistically significant pooled effect favouring telmisartan (Figure S3). For GGT, leave‐one‐out sensitivity analysis showed that the pooled estimate remained statistically significant after sequential exclusion of S. Alam et al. [15], S. Alam et al. [14], and Hirata et al. [16]. Exclusion of Georgescu et al. [21] resulted in a non‐significant pooled effect, although the direction of the association remained unchanged (Figure S4).

### 3.5 Publication Bias

Publication bias was assessed using funnel plots for outcomes with ≥ 5 studies. For outcomes with fewer than five studies, funnel plots and other statistical tests were not feasible due to insufficient data. Visual inspection of funnel plots suggested possible asymmetry across some outcomes; however, with five to six studies per outcome, these plots have insufficient power to reliably detect or exclude publication bias and should not be over‐interpreted. (Figures S5–S10).

## 4 Discussion

In this systematic review and meta‐analysis of randomized controlled trials, telmisartan did not demonstrate consistent improvements in metabolic, biochemical, or histological outcomes in patients with MASLD. Although fasting blood glucose showed a statistically significant reduction, the substantial heterogeneity and modest effect size limit confidence in the clinical relevance of this finding. Likewise, no significant improvements were observed in body mass index, waist circumference, insulin resistance, lipid profile, liver enzymes (except GGT), or histological outcomes. Overall, the current evidence does not support a consistent metabolic or hepatic benefit of telmisartan in MASLD. These patterns are best viewed as hypothesis‐generating rather than as proof of efficacy. BMI and waist circumference (WC) also did not improve significantly. Given that MASLD shares underlying pathophysiology with hypertension, dysglycemia, kidney vulnerability, and cardiovascular risk, these results are interpreted within the Cardiovascular Kidney Metabolic Syndrome (CKMS) framework [22]. An important consideration when interpreting these findings is the clinical heterogeneity of the comparator interventions. While some trials compared telmisartan with placebo or lifestyle modification, others used active comparators, including vitamin E, losartan, valsartan, and orlistat‐containing regimens. These interventions differ in their established metabolic and hepatic effects and may therefore have influenced the magnitude of the pooled treatment effects. Consequently, the observed benefits should be interpreted as relative to the specific comparator used rather than as an absolute estimate of telmisartan efficacy. Several factors likely contributed to the substantial heterogeneity observed across some pooled outcomes. The included trials differed considerably in their comparator interventions (placebo, lifestyle modification, vitamin E, losartan, valsartan, and orlistat‐containing regimens), follow‐up duration, baseline glycemic status, disease severity, and background treatments. These clinical and methodological differences may have influenced the magnitude of treatment effects and limit the certainty and generalizability of the pooled estimates. Consequently, outcomes with substantial heterogeneity should be interpreted cautiously.

ALT is widely used in clinical practice as a marker of hepatocellular injury in MASLD, despite its imperfect correlation with histology. Practice guidance emphasizes that aminotransferases can underestimate disease severity, yet ALT still remains a practical marker for monitoring treatment response when interpreted alongside metabolic context and noninvasive assessments [23]. In our analysis, ALT showed a favourable but non‐significant trend, which is biologically plausible given telmisartan's anti‐inflammatory and anti‐oxidative signalling effects through angiotensin II Type 1 receptor blockade and its metabolic actions through PPAR‐γ modulation. The absence of statistical significance may reflect the small sample sizes and short follow‐up durations across included trials. Clinical trials of telmisartan in NAFLD and NASH have reported reductions in ALT alongside improvements in steatosis markers and metabolic indices, suggesting that longer or larger trials may be needed to demonstrate a definitive effect [15, 24]. However, these findings from individual studies were not consistently confirmed in the pooled analysis, indicating that current clinical evidence remains insufficient to support a meaningful effect of telmisartan on liver enzyme improvement.

AST similarly did not show significant improvement. This pattern is not unexpected in MASLD because ALT often changes earlier and more noticeably than AST, while AST may be less responsive in earlier disease stages and may shift more clearly in advanced fibrosis or broader hepatocellular remodelling. Previous telmisartan studies also show variable AST responses, which may reflect baseline disease stage, duration of therapy, and differences in comparators and background lifestyle interventions [14, 15, 16].

Insulin resistance is a driver of MASLD and is also a core element of the cardiovascular kidney. Metabolic syndrome (CKMS) continuum. Although telmisartan has been proposed to improve insulin sensitivity through partial PPAR‐γ activation, the available randomized evidence did not demonstrate a significant improvement in HOMA‐IR. Therefore, the hypothesised metabolic mechanism has not translated into consistent clinical benefit. In a blinded comparative trial in hypertension‐associated NASH, telmisartan improved insulin resistance and histologic outcomes more than valsartan, suggesting that not all angiotensin receptor blockers are metabolically equivalent. Similarly, the FANTASY trial in hypertensive NAFLD with Type 2 diabetes reported improvement in liver steatosis metrics and insulin resistance markers with telmisartan compared with losartan [16].

Fasting glucose showed variable behaviour across studies, which is consistent with the fact that fasting glucose is influenced by baseline diabetic status, background glucose lowering therapy and trial duration. Although FBS reached statistical significance, substantial heterogeneity limits the reliability of this pooled estimate and suggests that the effect may not be consistent across patient populations and study designs. Thus, these results should be interpreted with caution. The available evidence does not show a consistent benefit of telmisartan on glycemic control. The observed reduction in fasting glucose should be seen as exploratory until it is confirmed in larger, similar randomized trials.

Telmisartan was associated with a statistically significant increase in GGT, which is the only adverse significant finding in this analysis and warrants direct discussion rather than dismissal. Whether a rise of this magnitude is clinically meaningful is debatable: a + 5.40 U/L increase falls within the normal laboratory reference range for most patients and is smaller than the fluctuations commonly seen with concurrent illness or dietary change. Nevertheless, it cannot be disregarded. Several biological mechanisms may account for this finding. GGT is sensitive to even low‐level alcohol consumption, and trials in this field typically permit alcohol intake up to accepted thresholds rather than requiring full abstinence; uneven distribution of drinking habits between treatment groups could introduce this imbalance [25, 26]. GGT also rises with cholestatic states and with certain medications, including some antihypertensives [27]. Beyond this, the comparator therapy also matters: in several included trials, the control group received lifestyle modification or placebo, whereas GGT may have fallen in those groups from dietary improvement, making telmisartan relatively worse. Baseline GGT values and their comparison between groups were not uniformly reported across included studies, limiting our ability to determine whether this finding reflects a true drug effect or a difference in baseline characteristics. In future trials, interpretation of GGT would be strengthened by reporting alkaline phosphatase and bilirubin trends, quantification of alcohol intake, and transparent concomitant medications usage [28].

Lipid indices are not primary hepatic activity outcomes in MASLD compared with histologic outcomes. Activity and fibrosis, yet they remain clinically important because cardiovascular disease is the leading cause of death in this population, which is central to the CKMS concept. In our pooled. Results, changes in LDL and HDL were not clearly significant, and triglycerides (TGs) showed a direction toward improvement without consistent statistical certainty. This pattern is consistent with prior telmisartan NAFLD trials and related studies, where lipid responses were modest and heterogeneous. The key clinical point is that telmisartan's value in MASLD is less about lipid lowering and more about integrated cardiometabolic risk control, including blood pressure, insulin resistance, vascular inflammation, and kidney protection. The current analysis does not demonstrate clinically meaningful effects of telmisartan on lipid parameters. Any broader cardiometabolic advantages of telmisartan derive from evidence outside the MASLD literature rather than from the present analysis.

Two outcomes that are particularly informative in our analysis are BMI and waist circumference (WC). The absence of changes in BMI and waist circumference indicates that no clinically meaningful effect on body composition was observed. Whether telmisartan exerts weight‐independent metabolic effects cannot be determined from the available evidence. Steatosis and inflammatory activity are core components of disease assessment in NASH, and improvement in these parameters is clinically meaningful when it reflects reduced hepatocyte. Injury and inflammatory signalling [29]. In our pooled analysis, neither the overall NAS nor its individual components (steatosis, ballooning, lobular inflammation) reached statistical significance. Although individual trials have reported histological improvements, the pooled evidence did not demonstrate consistent histological benefit, and therefore current clinical evidence remains insufficient to support such an effect. Importantly, these histological analyses were based on only a small number of randomized trials, limiting the precision and generalizability of the pooled estimates. Consequently, the available evidence is insufficient to draw firm conclusions regarding the histological efficacy of telmisartan in MASLD, and these findings should be regarded as hypothesis‐generating. This pattern is consistent with randomized trial evidence showing histologic benefit with telmisartan over longer follow‐up, including improvements in NAS components and fibrosis in biopsy confirmed NASH. The lack of clear statistical significance for ballooning and lobular inflammation in pooled analyses can still be compatible with a small treatment effect, particularly when baseline ballooning is mild, sample sizes are small, and the comparator includes lifestyle interventions that independently reduce inflammatory activity. These outcomes often require larger trials of longer follow‐up duration to demonstrate differences, especially when the expected effect is modest.

Among hepatic outcomes, fibrosis has the strongest prognostic significance for long‐term liver related events. In our analysis, fibrosis showed a mild, favourable trend that did not reach statistical significance. The pooled data do not support a conclusion of antifibrotic efficacy. This observation makes sense mechanistically, since angiotensin II signalling promotes hepatic stellate cell activation, oxidative stress, and fibrogenic signalling through AT1R, and blocking this receptor can limit these processes. Preclinical and translational studies have reported reductions in hepatic inflammation and fibrogenic markers with telmisartan, including modulation of inflammatory pathways and downregulation of fibrosis related pathways. One included RCT comparing telmisartan with valsartan reported more favourable histological outcomes with telmisartan, suggesting that its PPAR‐γ activity may add benefit beyond AT1R blockade alone; however, this cannot be confirmed from the current pooled analysis, given the non‐significant fibrosis result and small number of available studies [30]. Our findings are generally consistent with previous meta‐analyses evaluating angiotensin receptor blockers in NAFLD, which reported modest metabolic benefits but no consistent improvements across liver‐related outcomes. However, unlike these earlier class‐level analyses, our review focused exclusively on telmisartan and incorporated histological outcomes from randomized controlled trials [17]. This treatment‐specific approach provides a more focused assessment of telmisartan while also highlighting the limited evidence currently available for histological efficacy.

The overall pattern of results fits the view that MASLD is a multisystem disease driven by insulin resistance, adipose dysfunction, oxidative stress, inflammatory signalling and fibrogenesis, rather than a liver isolated process. Telmisartan potentially modifies several of these drivers at once. First, angiotensin II Type 1 receptor blockade reduces inflammatory and oxidative stress signalling that contributes to hepatocyte injury and stellate cell activation. Next, selective PPAR‐γ modulation improves insulin sensitivity and lipid partitioning, which can reduce lipotoxicity and downstream inflammation. Third, telmisartan's proven cardiovascular and renal outcome profile provides an important clinical anchor for its use in patients with MASLD who often have hypertension, high vascular risk, and early kidney vulnerability. Although these mechanisms provide a compelling biological rationale, the current pooled clinical evidence does not demonstrate that these theoretical effects translate into consistent metabolic or histological benefits in patients with MASLD. In ONTARGET, telmisartan was comparable to ramipril for major vascular outcomes and had a tolerability profile supportive of long‐term use [31]. Renal outcomes analyses in the same program show that telmisartan has similar major renal outcome effects to ramipril in high‐risk populations, which is relevant because kidney disease and metabolic risk interact within the cardiovascular kidney metabolic syndrome framework [32]. Accordingly, based on the current evidence, telmisartan should not be regarded as a disease‐modifying therapy for MASLD. Rather, its greatest clinical value may lie as a preferred antihypertensive agent for patients with MASLD who also require blood pressure control and have elevated cardiovascular or renal risk within the CKMS framework [22, 31, 32].

The GRADE assessment further supports cautious interpretation of our findings. Overall, the certainty of evidence ranged from low to moderate across evaluated outcomes, with downgrading primarily driven by inconsistency resulting from substantial between‐study heterogeneity and imprecision due to the limited number of available trials. Consequently, although some outcomes showed favourable numerical trends, the overall certainty of the evidence limits confidence in these estimates and underscores the need for larger, well‐designed randomized controlled trials before definitive clinical recommendations can be made.

### 4.1 Limitations

Several limitations of this review must be acknowledged. First, only six randomized controlled trials met the eligibility criteria, limiting the statistical power of the analyses. Although imaging‐based steatosis markers and non‐invasive fibrosis measures were pre‐specified as secondary outcomes, these endpoints were reported too inconsistently across the included studies to permit quantitative synthesis. The included studies also differed substantially in their comparator interventions, which ranged from placebo and lifestyle modification to active therapies such as vitamin E, losartan, valsartan, and orlistat‐containing regimens. This clinical heterogeneity may have influenced the pooled effect estimates and should be considered when interpreting the findings. Because very few studies were available within each comparator category, subgroup analyses or meta‐regression according to comparator type, diabetes status, diagnostic method, dose, and trial duration were not statistically feasible despite being pre‐specified in the protocol. Consequently, the sources of heterogeneity observed for several outcomes could not be fully explored. Grey literature was not searched, raising the possibility of publication bias. Funnel plots were generated for outcomes with five or more studies; however, with this number of studies, they lack sufficient power to detect publication bias reliably, and their visual interpretation is therefore limited; publication bias cannot be formally excluded. In addition, adverse events were inconsistently reported across the included trials, precluding a comprehensive assessment of the safety profile of telmisartan in patients with MASLD. The evidence base currently consists of short‐duration trials with modest sample sizes, and robust conclusions about efficacy or safety of telmisartan in MASLD will require larger, adequately powered RCTs with pre‐specified endpoints and longer follow‐ups.

## 5 Conclusion

Even though there is a convincing biological basis for RAAS inhibition and partial PPAR‐γ activation, existing clinical data does not show consistent improvements in metabolic, biochemical, or histological outcomes from telmisartan in MASLD. The slight decrease in fasting blood glucose noted is overshadowed by significant variability across studies and unclear clinical relevance. As a result, the current evidence does not indicate a clinically significant metabolic benefit of telmisartan beyond its recognized role in lowering blood pressure. To establish whether telmisartan provides any meaningful metabolic advantages for patients with MASLD, it is essential to conduct larger, well‐designed randomized controlled trials with standardized comparison treatments and extended follow‐up periods.

## Author Contributions

**Salma Nozhat:** investigation, validation, supervision. **Dorsa Shekouh:** conceptualization, writing – original draft, writing – review and editing, supervision. **Seyed Alireza Mirhosseini:** data curation, supervision, project administration, writing – review and editing. **Salar Azadnik:** writing – original draft, visualization, writing – review and editing. **Mahsa Borjzadehgashtaseb:** writing – original draft. **Asma Mousavi:** conceptualization, funding acquisition, validation, writing – review and editing. **Shayan Shojaei:** conceptualization, methodology, investigation. **Saeed Karami:** software, formal analysis, project administration, writing – original draft. **Salma Rostamipourfard:** writing – review and editing, project administration, formal analysis. **Ali Fatehi Hassanabad:** supervision. **Noureen Fatima:** validation, data curation, supervision, visualization, writing – review and editing. **Erfan Hashemi:** formal analysis, software, data curation, project administration. **Alireza Arzhangzadeh:** supervision, conceptualization, investigation, methodology.

## Funding

The authors have nothing to report.

## Ethics Statement

The authors have nothing to report.

## Conflicts of Interest

The authors declare no conflicts of interest.

## Supporting information


**Figure S1:** Leave‐one‐out sensitivity analysis for HOMA‐IR change studies.
**Figure S2:** Leave‐one‐out sensitivity analysis for FBS change studies.
**Figure S3:** Leave‐one‐out sensitivity analysis for the NAFLD activity score (NAS).
**Figure S4:** Leave‐one‐out sensitivity analysis for GGT change studies.
**Figure S5:** Funnel plot assessing publication bias in BMI change studies.
**Figure S6:** Funnel plot assessing publication bias in WC change studies.
**Figure S7:** Funnel plot assessing publication bias in FBS change studies.
**Figure S8:** Funnel plot assessing publication bias in TG change studies.
**Figure S9:** Funnel plot assessing publication bias in HDL change studies.
**Figure S10:** Funnel plot assessing publication bias in ALT change studies.
**Table S1:** Database search queries and strategy.
**Table S2:** Risk of bias assessment of included studies using the RoB2 tool.
**Table S3:** GRADE certainty of evidence assessment.

## Data Availability

Data sharing not applicable to this article as no datasets were generated or analysed during the current study.
