# Supplementary material for: Efficacy of Telmisartan in MASLD/NAFLD: A Systematic Review and Meta‐Analysis of Randomized Controlled Trials
Source: Endocrinol Diabetes Metab. 2026 Aug 19;9(5):e70319. doi: 10.1002/edm2.70319 (PMC13487458; doi:10.1002/edm2.70319)
Supplement: Supplementary file 1 [file EDM2-9-e70319-s001.docx]

# Supplementary Materials

*Supplementary Figure 1. Leave-one-out sensitivity analysis for HOMA-IR change studies*

*Supplementary Figure 2. Leave-one-out sensitivity analysis for FBS change studies*

*Supplementary Figure 3. Leave-one-out sensitivity analysis for the NAFLD activity score (NAS)*

*Supplementary Figure 4. Leave-one-out sensitivity analysis for GGT change studies*

*Supplementary Figure 5. Funnel plot assessing publication bias in BMI change studies*

*Supplementary Figure 6. Funnel plot assessing publication bias in WC change studies*

*Supplementary Figure 7. Funnel plot assessing publication bias in FBS change studies.*

*Supplementary Figure 8. Funnel plot assessing publication bias in TG change studies.*

*Supplementary Figure 9. Funnel plot assessing publication bias in HDL change studies.*

*Supplementary Figure 10. Funnel plot assessing publication bias in ALT change studies.*

*Supplementary Table 1. Database search queries and strategy***.**

| Database |  |
| --- | --- |
| Pubmed | ("Telmisartan"[Mesh] OR telmisartan[All Fields] OR micardis[All Fields] OR "angiotensin II receptor blocker"[All Fields] OR "AT1 receptor blocker"[All Fields] OR ARB[All Fields] OR "PPAR-gamma modulator"[All Fields] OR "PPAR gamma modulator"[All Fields] OR "renin-angiotensin system inhibitor"[All Fields] OR "renin angiotensin system inhibitor"[All Fields] OR "RAAS blockade"[All Fields] OR antihypertensive[All Fields])  AND  ("Fatty Liver"[Mesh]OR "non-alcoholic fatty liver disease"[All Fields]OR "non alcoholic fatty liver disease"[All Fields]OR NAFLD[All Fields]OR NASH[All Fields]OR "fatty liver"[All Fields]OR steatosis[All Fields]OR "non-alcoholic steatohepatitis"[All Fields]OR "non alcoholic steatohepatitis"[All Fields]OR steatohepatitis[All Fields]OR "metabolic associated fatty liver disease"[All Fields]OR MAFLD[All Fields]OR "metabolic dysfunction-associated steatotic liver disease"[All Fields]OR MASLD[All Fields]) |
| Scopus | (TITLE-ABS-KEY(telmisartan)OR TITLE-ABS-KEY(micardis)OR TITLE-ABS-KEY("angiotensin II receptor blocker")OR TITLE-ABS-KEY("AT1 receptor blocker")OR TITLE-ABS-KEY(ARB)OR TITLE-ABS-KEY("PPAR-gamma modulator")OR TITLE-ABS-KEY("PPAR gamma modulator")OR TITLE-ABS-KEY("renin-angiotensin system inhibitor")OR TITLE-ABS-KEY("renin angiotensin system inhibitor")OR TITLE-ABS-KEY("RAAS blockade")OR TITLE-ABS-KEY(antihypertensive))  AND  (TITLE-ABS-KEY("non-alcoholic fatty liver disease")OR TITLE-ABS-KEY("non alcoholic fatty liver disease"OR TITLE-ABS-KEY(NAFLD)OR TITLE-ABS-KEY(NASH)OR TITLE-ABS-KEY("fatty liver")OR TITLE-ABS-KEY(steatosis)OR TITLE-ABS-KEY("non-alcoholic steatohepatitis")OR TITLE-ABS-KEY("non alcoholic steatohepatitis")OR TITLE-ABS-KEY(steatohepatitis)OR TITLE-ABS-KEY("metabolic associated fatty liver disease")OR TITLE-ABS-KEY(MAFLD)OR TITLE-ABS-KEY("metabolic dysfunction-associated steatotic liver disease")OR TITLE-ABS-KEY(MASLD)) |
| Cochrane | (MeSH descriptor: [Telmisartan] explode all trees OR telmisartan:ti,ab,kw OR Micardis:ti,ab,kw OR "angiotensin II receptor blocker":ti,ab,kw OR "AT1 receptor blocker":ti,ab,kw OR ARB:ti,ab,kw OR "PPAR-gamma modulator":ti,ab,kw OR "PPAR gamma modulator":ti,ab,kw OR "renin-angiotensin system inhibitor":ti,ab,kw OR "renin angiotensin system inhibitor":ti,ab,kw OR "RAAS blockade":ti,ab,kw OR antihypertensive:ti,ab,kw  ) AND (MeSH descriptor: [Fatty Liver] explode all trees OR "non-alcoholic fatty liver disease":ti,ab,kw OR "non alcoholic fatty liver disease":ti,ab,kw OR NAFLD:ti,ab,kw OR NASH:ti,ab,kw OR "fatty liver":ti,ab,kw OR steatosis:ti,ab,kw OR "non-alcoholic steatohepatitis":ti,ab,kw OR "non alcoholic steatohepatitis":ti,ab,kw OR steatohepatitis:ti,ab,kw OR "metabolic associated fatty liver disease":ti,ab,kw OR MAFLD:ti,ab,kw OR "metabolic dysfunction-associated steatotic liver disease":ti,ab,kw OR MASLD:ti,ab,kw) |
| Embase | (telmisartanOR micardisOR "angiotensin II receptor blocker"OR "AT1 receptor blocker"OR ARBOR "PPAR-gamma modulator"OR "PPAR gamma modulator"OR "renin-angiotensin system inhibitor"OR "renin angiotensin system inhibitor"OR "RAAS blockade"OR antihypertensive)  AND  ("non-alcoholic fatty liver disease"OR "non alcoholic fatty liver disease"OR NAFLDOR NASH OR "fatty liver"OR steatosisOR "non-alcoholic steatohepatitis"OR "non alcoholic steatohepatitis"OR steatohepatitisOR "metabolic associated fatty liver disease"OR MAFLDOR "metabolic dysfunction-associated steatotic liver disease"OR MASLD) |

*Supplementary Table 2. Risk of bias assessment of included studies using the RoB2 tool*

| First author/  Year | Domain 1 | Domain 2 | Domain 3 | Domain 4 | Domain 5 | Overall |
| --- | --- | --- | --- | --- | --- | --- |
| VAW. Esmail  2022 | Low | Low | Low | Low | Low | Low |
| S. Alam  2020 | Low | Low | Low | Low | Low | Low |
| MSM. Al-Nimer  2019 | Low | Low | Low | Low | Low | Low |
| S. Alam  2016 | Low | Some concern | Low | Low | Low | Some concern |
| T. Hirata  2013 | Some concern | Low | Low | Low | Low | Some concern |
| EF. Georgescu  2009 | Low | Low | Low | Low | Low | Low |

### *Supplementary Table 3. GRADE certainty of evidence assessment*

| **Outcome** | **Studies (n)** | **Effect Estimate (MD, 95% CI)** | **Risk of Bias** | **Inconsistency** | **Indirectness** | **Imprecision** | **Publication Bias** | **Certainty of Evidence** |
| --- | --- | --- | --- | --- | --- | --- | --- | --- |
| Fibrosis | 3 | -0.28 (-0.57 to 0.01) | Not serious | Not serious | Not serious | Serious | Undetected | ⨁⨁⨁◯ Moderate |
| NAS | 3 | -0.38 (-1.70 to 0.94) | Not serious | Very serious | Not serious | Serious | Undetected | ⨁⨁◯◯ Low |
| FBS | 5 | -2.78 (-5.17 to -0.39) | Not serious | Very serious | Not serious | Serious | Suspected | ⨁⨁◯◯ Low |
| ALT | 5 | -1.37 (-6.72 to 3.98) | Not serious | Not serious | Not serious | Serious | Suspected | ⨁⨁⨁◯ Moderate |
| HOMA-IR | 4 | -0.40 (-1.43 to 0.63) | Not serious | Very serious | Not serious | Serious | Undetected | ⨁⨁◯◯ Low |
| BMI | 6 | 0.23 (-0.07 to 0.53) | Not serious | Not serious | Not serious | Serious | Suspected | ⨁⨁⨁◯ Moderate |
| GGT | 4 | 5.40 (0.51 to 10.29) | Not serious | Not serious | Not serious | Serious | Undetected | ⨁⨁⨁◯ Moderate |

GRADE Working Group grades of evidence

High certainty (⨁⨁⨁⨁): very confident that the true effect lies close to that of the estimate.

Moderate certainty (⨁⨁⨁◯): moderately confident in the effect estimate; the true effect is likely close to the estimate but may be substantially different.

Low certainty (⨁⨁◯◯): limited confidence in the effect estimate; the true effect may be substantially different from the estimate.
